# Quality-of-Life Evaluation in Coeliac Patients on a Gluten-Free Diet

**DOI:** 10.3390/nu12102981

**Published:** 2020-09-29

**Authors:** Ilaria Marsilio, Cristina Canova, Anna D’Odorico, Matteo Ghisa, Letizia Zingone, Greta Lorenzon, Edoardo Vincenzo Savarino, Fabiana Zingone

**Affiliations:** 1 Department of Surgery, Oncology and Gastroenterology, Gastroenterology Section, University Hospital of Padua, 35128 Padua, Italy; ilaria.marsilio@gmail.com (I.M.); annadodorico@unipd.it (A.D.); matteoghisa@yahoo.it (M.G.); gretalorenzon90@gmail.com (G.L.); edoardo.savarino@unipd.it (E.V.S.); 2 Department of Cardiac, Thoracic and Vascular Sciences and Public Health, University Hospital of Padua, 35128 Padua, Italy; cristina.canova@unipd.it; 3 Independent Researcher, 80128 Naples, Italy; letiziazingone@libero.it

**Keywords:** coeliac disease, quality of life, gluten-free diet

## Abstract

The treatment for coeliac disease (CD) has a considerable psychological impact on patients, which may vary depending on subjects and clinical characteristics. The aim of this study was to describe the quality of life (QoL) in CD patients during follow-up, evaluating which factors can influence it. Patients with CD who consecutively visited the outpatient clinic of CD Unit of the University Hospital of Padua from January to September 2019 were enrolled. Demographics and clinical information were collected, and all patients were asked to answer the CD-QoL and Biagi’s validated questionnaires. Student’s *t*-test and chi-square test were used to compare the continuous and categorical variables, respectively. One hundred patients were enrolled (86 females, mean age at test ± SD: 39.73 ± 13.51; mean age at diagnosis ± SD: 33.09 ± 12.92), with 61% of them having been diagnosed with CD within the previous 5 years. At the time of diagnosis, 43 CD patients reported classical CD presentation, 32 non-classical features, 16 only anaemia and 9 were asymptomatic. The mean CD-QoL value was overall high (80.54 ± 11.91). We found that the “health concerns” subscale score was significantly lower in subjects aged more than 35 years compared to younger subjects (*p* = 0.03). We also observed that the CD-QoL score in gluten-free diet (GFD)-adherent patients tended to be higher compared to subjects who were non-compliant, with a significantly higher percentage of patients with low score for the “dysphoria” subscale (*p* = 0.05). This study showed an overall good QoL in subjects on a GFD. However, subjects older and non-compliant to GFD appear to experience more health concerns and suffer from dysphoria, respectively.

## 1. Introduction

Coeliac disease (CD) is a permanent intolerance to dietary gluten [1]; therefore, ingestion of this protein induces an abnormal immunological response in genetically predisposed subjects, resulting in intestinal inflammation, followed by the atrophy of villi, crypt hyperplasia and increased infiltration by intraepithelial lymphocytes [2]. The prevalence of CD has significantly increased over the past 10–25 years and is around 1% in the population worldwide, with a female predominance [3,4,5]. CD is heavily underdiagnosed, partially due to the extensive clinical signs and symptoms. Gastrointestinal (GI) symptoms and signs of malabsorption represent the most common clinical presentations of CD [6]; however, patients may experience various extra-intestinal symptoms and multi-organ manifestations, reflecting the systemic nature of the disease [7]. The treatment for CD is primarily a lifelong strict gluten-free diet (GFD), which arrests autoimmune response, allowing mucosal healing [3]. A low adherence to a GFD exposes the patient to complications and morbidity [8]. Unfortunately, the strict adherence to diet implies serious abandonments and lifestyle changes, which directly affect the quality of life (QoL) [9]. A recent literature review has described the burden experienced by adult CD patients in eating away from home, reading food labelling to avoid inadvertent gluten exposure, travelling and having meals with other people [10]. Therefore, CD patients tend to think about their diet and can feel negative emotions such as anxiety, fear and frustration, and avoid social activities. The health-related QoL investigated in patients with CD showed slightly lower scores compared to healthy controls [11]. Besides diet, other factors have also been associated with worse QoL in CD patients on a GFD such as clinical presentation, age at diagnosis and gender [12,13,14,15]. Since the available studies are mainly based on the use of generic questionnaires on the QoL, we aimed to investigate the QoL in a cohort of CD patients on a GFD, using a validated CD-specific questionnaire that refers to the impact of the disease on the individual’s QoL, further evaluating potential factors, such as clinical presentation, dietary adherence, age of diagnosis and time of diagnosis, that may influence it.

## 2. Material and Methods

We consecutively and prospectively recruited all adult CD patients who visited the CD Unit of the University Hospital of Padua (Department of Surgery, Oncology and Gastroenterology, Gastroenterology Section, Italy) from January to September 2019. The inclusion criteria were age ≥18 years and a CD diagnosis, from at least 1 year, based on serology and histological evaluation [3]. Exclusion criteria were the inability to complete questionnaires, significant psychiatric diagnoses (including dementia) and refusal to sign the informed consent form. We also excluded patients with known refractory CD type 1 or 2. All participants were informed about the nature, duration and purpose of the study. The study was approved by the research ethics committee of the University Hospital of Padua (CESC 4680/AO/19). At the time of enrolment, subjects’ demographic data (age and gender) and clinical characteristics such as date of CD diagnosis, serology and histology at CD diagnosis, signs and symptoms at CD diagnosis and follow-up and values of antibody anti-transglutaminase IgA (tTG-IgA) at follow-up were collected. All subjects were asked to complete the CD-specific Quality of Life Scale (CD-QoL), a specific instrument that refers to the impact of CD on the individual’s QoL (Appendix A) [16]. The Italian CD-QoL questionnaire a validated tool to assess health-related QoL [17], consists of 20 questions across 4 clinically relevant dimensions (CD-related limitations, dysphoria, health concerns and inadequate treatment). Each item was scored on a 5-point Likert scale, and total scores ranged between 20 and 100, with higher scores corresponding to better QoL. To date, no clear cut-off point has been established to dichotomize CD-QoL scores. We divided the score in three tertiles, and patients in the lowest tertile were considered to have a poorer QoL. In addition, the level of adherence to GFD was assessed using Biagi’s questionnaire, a validated tool to evaluate dietary compliance, which consists of a few questions in the Italian language that can be administered in a few minutes even by non-expert personnel [18]. The numerical result makes it possible to monitor the strictness of GFD compliance over time. The questionnaire is drawn up in the form of an algorithm, and the final score is made up of five levels (0–4), which, from a clinical point of view, can be grouped into three levels. Patients with a score of 0 or 1 do not follow a strict GFD, whereas patients with a score of 2 follow a GFD but with important errors that require correction. Patients with a score of 3 or 4 follow a strict GFD.

### Statistical Analysis

Continuous variables were expressed as means ± standard deviation (SD), while categorical data were given as counts and percentages. Chi-square and Student’s *t*-test were used for the analysis of categorical and continuous data, respectively. STATA 11 software was used for statistical analyses (Stata Corp., College Station, TX, USA).

## 3. Results

### 3.1. Study Population

We targeted all patients who visited our unit during the study period. However, seven patients refused to be included due to lack of time, two were excluded because they were unable to complete the questionnaire due to a language barrier, three because they were diagnosed with refractory CD and fifteen because they had been on a GFD for less than one year. As presented in Table 1, 100 CD patients were finally enrolled (86 females and 14 males) with a mean age of 39.73 (13.51) years at the time of the study and an average of 6.64 (6.39) years from CD diagnosis. The mean age at CD diagnosis was 33.09 (12.92) years. All participants met the diagnostic criteria according to current guidelines [3]. At the time of diagnosis, 43 patients had classical symptoms (i.e., diarrhoea and weight loss), 32 had non-classical features (i.e., abdominal pain, constipation, asthenia), 16 had anaemia only and 9 were asymptomatic. At the time of diagnosis, two patients were seronegative with villous atrophy (Marsh 3), and histological remission was seen after GFD. The mean follow-up was 6.64 (6.39) years. At the time of follow-up, all participants reported the normalization of CD serology, with the exception of 15 patients who presented a slight increase of antibody tTG-IgA with a mean value of 22.85 kU/L (normal limit < 7). Based on Biagi’s questionnaire, most of the patients (n = 90) reported strict adherence to a GFD, five were judged adherent to a GFD but with important errors that required some changes, and five did not adhere to a GFD.

Table 1 presents the demographics and clinical characteristics of the participants. Of the 15 patients with positive antibody tTG-IgA at the follow-up, all showed a score of 3 or 4 on Biagi’s questionnaire, indicating a strict GFD adherence.

### 3.2. QoL Questionnaire

**Figure 1:**
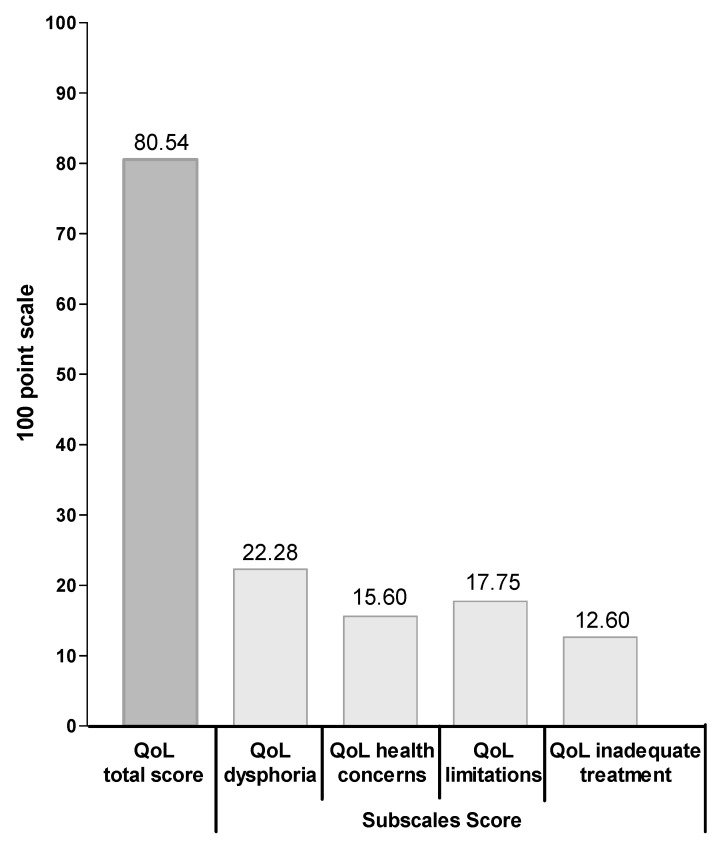
CD-specific Quality of Life Scale (CD-QoL) score and subscale scores.

The mean CD-QoL total score was 80.54 (11.91). In detail, the “dysphoria” subscale score was 22.28 (3.25), the “health concerns” subscale score was 15.60 (3.34), the “limitation” subscale score was 17.75 (3.89) and the “inadequate treatment” subscale score was 12.60 (2.60) (Figure 1). We divided the CD-QoL total score and each sub-score in three tertiles to better stratify the correlation between each score and independent variables including the time of diagnosis, age at diagnosis, Biagi’s score (GFD adherence), gender and type of clinical presentation. Thirty-five patients had a CD-QoL total score in the first tertile (range 44–77), thirty-three in the second tertile (range 78–88) and thirty-two in the third tertile (range 89–99). We found that the “health concerns” subscale score was significantly lower in subjects aged more than 35 years compared to younger ones (*p* = 0.03, Table 2). We also observed that the CD-QoL total score in GFD-adherent patients tended to be higher compared to subjects who were non-compliant (*p* = 0.07, Table 2), with a significantly higher percentage of patients with low score for the “dysphoria” subscale (*p* = 0.05, Table 2). No statistically significant difference was observed according to gender and time from diagnosis (*p* > 0.05, Table 2).

## 4. Discussion

This cross-sectional evaluation showed a good overall QoL in CD patients on a GFD. However, subjects older and non-compliant to GFD appear to experience more health concerns and suffer from dysphoria, respectively. Previous literature reported that the QoL of a CD patient at diagnosis is mainly related to the presence of GI or non-GI symptoms, while after the diagnosis, the QoL is mainly associated to the difficulty of having a chronic condition and the limitations imposed by the GFD as well as the difficulties in maintaining a good diet compliance [19,20]. The negative psycho-social aspects of GFD, with its restrictions affecting daily life, represent a higher significant burden compared to those associated with other chronic diseases, including inflammatory bowel disease and type 1 diabetes [21]. The CD-QoL is a simple tool to understand the perception of limitations and difficulties in the everyday life of CD patients [17]. The benefit of using the CD-QoL questionnaire to evaluate the effect on QoL of CD diagnosis and GFD compared to generic QoL questionnaires is that the questions included are specific to the CD, the GFD and their limitations. Moreover, compared to the other CD-specific questionnaires, the Coeliac Disease Questionnaire ([22]) and Coeliac Disease Assessment Questionnaire ([23]), the CD-QoL is shorter and takes less time for patients to complete. This study indicates that the principal factors most affected by CD were health concerns and dysphoria. The perception of the health status in CD patients with age at diagnosis higher than 35 years was worse compared to the younger patients. The participants described an impact on their physical health, including concerns about the impact of CD on daily life, the risk of developing other health problems, the risk of cancer and the long-term health effects. This probably reflects the knowledge that older age is considered to be a risk factor for complicated CD [24,25]. Accordingly, some previous studies reported a negative correlation between QoL and age since older patients often report comorbidities and severe symptoms limiting their health [26,27]. Furthermore, elderly patients may find more difficult to follow a GFD due to several factors, such as the dependence on a partner orvisual impairment that impedes the reading of food labels. Moreover, those who live alone could easily feel discouraged by the thought of changing the dietary habits of a lifetime and be fearful of the cost implications and how products might be obtained [28].

To note, we have recently used the same CD-QoL questionnaire to evaluate the perceived impact of the COVID-19 pandemic on the QoL of our coeliac population, finding an impact particularly in women, elders and patients with other comorbidities. The total questionnaire score obtained during the COVID-19 pandemic (77.4 ± 10.03) resulted slightly lower than the results found in this study, which refers to a pre-pandemic period [29,30].

Additionally, in this study, we identified a considerable psychological impact on the emotional health of CD patients non-compliant to GFD, which may affect their social life in terms of relationships with people, state of isolation and depression. Previous studies have shown that poor dietary adherence is often associated with a poor QoL, but it is difficult to identify which is the cause and which is the effect [11]. Previous literature reported contradictory results regarding this association. Indeed, a long-term longitudinal study suggested that subsequent deterioration in QoL was associated with a lack of dietary adherence [31], while others reported no differences in QoL scores between patients with full adherence and patients with partial/non-adherence to GFD [32,33].

CD patients feel cautious when eating food that is not prepared at home. A survey published in 2006 and conducted on 2681 adult members of the Canadian Celiac Association showed that 44% of them found difficulties following the GFD, which included determining if foods were gluten free (85%), finding gluten-free foods in stores (83%), avoiding restaurants (79%) and avoiding travel (38%; [34]). Seven years later, another survey conducted in Canada among the same population showed that difficulties and negative emotions were experienced less frequently by those on the diet for >5 years, although food labelling and eating away from home remained very problematic [35]. Ford et al. reported that self-efficacy and illness perception might play a role in the individual’s psycho-education [36]. Furthermore, the vigilance required for a GFD may generate a fixation with food intake and fear of eating, and significantly increase the risk of developing eating disorders [37]. Therefore, a therapeutic intervention might improve GFD adherence and enhance the psychological well-being of CD patients.

We did not find a different QoL in patients with different clinical presentations at diagnosis. While previous literature mainly reported that the GFD induces an improvement in QoL in symptomatic patients, a similar effect has not been clearly reported in patients with subclinical/asymptomatic CD [13,14]. Paavola et al., analysing CD patients on long-term GFD, reported that the QoL was unimpaired in screen-detected coeliac patients and lower in symptom-detected patients, when compared to healthy controls [38]. We also did not identify a different QoL between males and females, while most previous studies reported a lower QoL in females than in males [15,39,40,41]. We included consecutive patients, and sex was not a selection criterion for the study. The prevalence of females in our overall study population is in line with the known predominance of females in CD. Moreover, as a tertiary referral centre, females with other autoimmune diseases and gynaecological problems, which could be related to CD, are all referred to us.

Regarding the age from diagnosis, the main reason for a 5-year cut-off is to align with previous studies, which had cut-offs of <1 year, 1 to 5 years or >5 years [35]. As we exclude patients who have been on a GFD for less than 1 year and, according to Zarkadas et al., difficulties and negative emotions were experienced less frequently by those on the diet for >5 years, we decided to use the 5-year cut-off.

The Biagi score is a self-reported questionnaire that did not necessarily reflect the real daily gluten intake; in fact, we have 15 patients who presented a slight increase of antibody tTG-IgA while reporting a strict adherence to a GFD. Recently in the literature, a new strategy to improve the identification of GFD non-compliance was reported as the quantification of faecal gluten immunogenic peptides [42]. The 15 subjects reported a significant reduction of their antibodies after six months from the dietician consultation we suggested.

In this study, we offered a real-life evaluation of the QoL of CD patients through a standardized questionnaire, in a sample of patients followed in a tertiary outpatients’ referral centre. This might be a possible limitation in terms of the generalizability of the results since CD patients struggle to return to follow-up if they feel good. Moreover, the cross-sectional nature of the study did not allow us to evaluate the change over time of the impact of the CD diagnosis on the QoL, as previously done in [31].

In conclusion, taking into consideration these results, we believe that the CD-QoL questionnaire should be used in clinical practice to assess changes in the CD impact on QoL, identify any external contributing factors and arrange psychological support if required. In particular, we believe that older patients and non-GFD-compliant patients should be strictly followed and supported to improve their QoL and disease perception.

The potential benefit of this approach in the management of CD patients should be evaluated in long-term prospective studies.

## Figures and Tables

**Table 1:** Study population characteristics.

Coeliac Disease Population Characteristic	
N°	100
Female gender	86
Age at time of testing (mean (SD))	39.73 (13.51)
Age at CD diagnosis, yrs (mean (SD)) Age at DIA ˃ 35 yrs (n° patients)	33.09 (12.92) 40
Yrs from DIA (mean (SD)) Yrs from DIA ˃ 5 yrs (n° patients)	6.64 (6.39) 39
CD presentation (n° patients)	
Classical CD Only anaemia Non-classical CD Asymptomatic	43 16 32 9
tTG-IgA antibody at DIA (n° patients)	
Negative Positive	2 98
tTG-IgA antibody at FU (n° patients)	
Negative Positive	85 15

Abbreviations: standard deviation, SD; coelic disease, CD; diagnosis, DIA; anti-transglutaminase IgA, tTG-IgA; follow-up, FU.

**Table 2:** CD-QoL scores stratified by time of diagnosis, age at diagnosis, Biagi score, gender and symptoms.

CD-QoL	Time DIA		*P* °	Age DIA		*P* °	Biagi Score		*P* °	Gender		*P*	Symptoms		*P* °
	≤5 yrs	>5 yrs		≤35 yrs	>35 yrs		Non-Adherent	Adherent		M	F		Other	Classic	
CD-QoL * total	80.7	80.3	0.88	81.5	79.1	0.32	73.7	81.2	0.07	80.6	80.5	0.97	81.3	79.5	0.46
Low	34.4%	35.9%	0.79	35%	35%	0.66	55.6%	33%	0.28	28.6%	36.1%	0.64	31.6%	39.5%	0.11
Medium	31.2%	35.9%		30%	37.5%		33.3%	33%		28.6%	33.7%		28.1%	39.5%	
High	34.4%	28.2%		35%	27.5%		11.1%	34%		42.8%	30.2%		40.3%	21%	
Dysphoria *	22.4	22.1	0.67	22.4	22.2	0.71	21.2	22.4	0.3	22.2	22.3	0.92	22.2	22.4	0.85
Low	36.1%	38.5%	0.55	35%	40%	0.76	66.7%	34%	**0.05**	28.6%	38.4%	0.46	35.1%	39.5%	0.55
Medium	31.2%	38.5%		33.3%	35%		0%	37.4%		28.6%	34.9%		31.6%	37.2%	
High	32.7%	23%		31.7%	25%		33.3%	28.6%		42.8%	26.7%		33.3%	23.3%	
Health concerns *	15.7	15.5	0.78	16.2	14.7	**0.03**	13.8	15.8	0.09	15.7	15.6	0.87	16.1	15	0.13
Low	39.3%	48.8%	0.62	35%	55%	0.13	55.6%	41.8%	0.10	57.1%	40.7%	0.19	36.8%	51.2%	0.35
Medium	27.9%	25.6%		30%	22.5%		44.4%	25.3%		7.2%	30.2%		29.8%	23.3%	
High	32.8%	25.6%		35%	22.5%		0%	32.9%		35.7%	29.1%		33.4%	25.5%	
Limitations *	17.8	17.6	0.87	17.8	17.5	0.68	16	17. 9	0.17	17.2	17.8	0.60	17.9	17.4	0.47
Low	36.1%	30.8%	0.25	35%	32.5%	0.96	44.4%	32.9%	0.73	42.9%	32.6%	0.61	29.8%	39.5%	0.25
Medium	27.8%	43.6%		33.3%	35%		33.3%	34.2%		35.7%	33.7%		31.6%	37.2%	
High	36.1%	25.6%		31.7%	32.5%		22.3%	32.9%		21.4%	33.7%		38.6%	23.3%	
Inadequate treatment *	12.5	12.8	0.51	12.7	12.5	0.64	11.8	12.7	0.32	12.9	12.6	0.61	12.6	12.5	0.83
Low	36.1%	33.4%	0.90	33.3%	37.5%	0.84	55.6%	33%	0.21	28.6%	36.1%	0.22	33.4%	37.3%	0.53
Medium	44.3%	48.7%		48.3%	42.5%		44.4%	46.2%		34.7%	47.7%		43.8%	48.8%	
High	19.6%	17.9%		18.4%	20%		0%	20.8%		35.7%	16.2%		22.8%	13.9%	

* Mean score value, ° Student’s *t*-test for comparing continuous variables and chi-square test for comparing categorical ones. Statistically significant *p*-values are indicated in bold.

## Appendix A. CD-QoL Scale

Please think about your life over the past month (30 days) and look at the statements below. Each statement has five possible responses. For each statement, please fill in one box in each row that best describes your feelings.

**Statements**	**Not at All**	**A Little**	**Enough**	**Much**	**Very Much**
1. I feel limited by this disease					
2. I feel worried that I will suffer from this disease					
3. I fell concerned that this disease will cause other health problems					
4. I feel worried about my increased risk of cancer from this disease					
5. I feel socially stigmatized for having this disease					
6. I feel like I’m limited in eating meals with co workers					
7. I feel like I am not able to have special foods like birthday cake and pizza					
8. I feel that the diet is insufficient treatment for my disease					
9. I feel that there are not enough choices for treatment					
10. I feel depressed because of my disease					
11. I feel frightened by having this disease					
12. I feel like I don’t know enough about the disease					
13. I feel overwhelmed about having this disease					
14. I have trouble socializing because of my disease					
15. I find it difficult to travel or take long trips because of my disease					
16. I feel like I cannot live a normal life because of my disease					
17. I feel afraid to eat out because my food may be contaminated					
18. I feel worried about the increased risk of one of my family members having celiac disease					
19. I feel like I think about food all the time					
20. I feel concerned that my long-term health will be affected					
